# Osteocalcin Alleviates Nonalcoholic Fatty Liver Disease in Mice through GPRC6A

**DOI:** 10.1155/2021/9178616

**Published:** 2021-01-15

**Authors:** Mingliang Zhang, Xiaomin Nie, Yeqing Yuan, Yansu Wang, Xiaojing Ma, Jun Yin, Yuqian Bao

**Affiliations:** Department of Endocrinology and Metabolism, Shanghai Jiao Tong University Affiliated Sixth People's Hospital, Shanghai Clinical Center for Diabetes, Shanghai Key Clinical Center for Metabolic Disease, Shanghai Diabetes Institute, Shanghai Key Laboratory of Diabetes Mellitus, Shanghai 200233, China

## Abstract

Osteocalcin is a bone-derived hormone that plays an important role in the crosstalk between bone and energy metabolism. Previous studies have found that treatment with uncarboxylated osteocalcin can protect mice from high-fat diet-induced nonalcoholic fatty liver disease (NAFLD). However, the potential mechanisms remain unclear. Although the G protein-coupled receptor family C group 6 subtype A (GPRC6A) is the putative receptor of osteocalcin, there is no direct evidence showing that GPRC6A mediates the effects of uncarboxylated osteocalcin in alleviating NAFLD in mice. We aimed to figure out this using liver-specific GPRC6A knockout (GPRC6A^LKO^) mice. Consistent with previous studies, uncarboxylated osteocalcin significantly protected high-fat diet-fed wild-type mice from obesity and NAFLD, while it did not protect high-fat diet-fed GPRC6A^LKO^ mice from NAFLD. Differential mRNA expression of lipogenesis and lipolysis between GPRC6A^LKO^ mice and control mice revealed that GPRC6A mediated the effects of osteocalcin in alleviating NAFLD through inhibiting lipid synthesis and promoting lipolysis. In conclusion, this study found that uncarboxylated osteocalcin alleviates NAFLD in mice through the GPRC6A signaling pathway. Our study suggests that liver GPRC6A may be a potential target for treating NAFLD.

## 1. Introduction

The prevalence of nonalcoholic fatty liver disease (NAFLD) is increasing rapidly worldwide [1]. NAFLD is regarded as the hepatic manifestation of metabolic syndrome and is frequently associated with abdominal obesity, hypertension, diabetes, and cardiovascular diseases [2]. The histological spectrum of NAFLD ranges from simple steatosis to nonalcoholic steatohepatitis [3]. An abundance of hormones and cytokines are involved in the pathogenesis of NAFLD [4].

Osteocalcin, a bone-derived hormone, is synthesized and secreted by osteoblasts. The vitamin-K-dependent *γ*-carboxylated form of osteocalcin regulates the size and shape of hydroxyapatite. In recent years, the extra-bone functions of osteocalcin have gained increasing attention [5, 6]. Epidemiological studies have found that serum total osteocalcin levels are inversely correlated with NAFLD [7], abdominal obesity [8], subclinical atherosclerosis [9], and metabolic syndrome [10]. Basic experiments have shown that the uncarboxylated form of osteocalcin increases pancreatic *ß*-cell proliferation, insulin release from pancreatic islets, insulin sensitivity, and energy expenditure [11]. Mice lacking osteocalcin display decreased pancreatic cell proliferation, glucose intolerance, and insulin resistance [11]. Moreover, treatment with uncarboxylated osteocalcin protects mice from diet-induced hepatic triglyceride accumulation [12, 13]. However, the mechanisms underlying the role of uncarboxylated osteocalcin in NAFLD remain unclear and need to be investigated.

G protein-coupled receptor family C group 6 subtype A (GPRC6A) is a master regulator of metabolism [14]. GPRC6A has been proposed to integrate metabolic functions through the coordinated secretion of hormones, including insulin, glucagon-like peptide-1, and interleukin 6, and the direct role of this receptor in controlling glucose and fat metabolism in the liver, skeletal muscle, and adipose tissues [15–18]. Several previous studies have reported that GPRC6A mediates the metabolic and endocrine effects of uncarboxylated osteocalcin. GPRC6A knockout in mice results in metabolic syndrome, and the activation of GPRC6A stimulates the proliferation of *ß*-cells, increases peripheral insulin sensitivity, and protects against high-fat diet (HFD)-induced metabolic abnormalities [14, 19, 20]. The intraperitoneal injection of osteocalcin increases circulating insulin levels in wild-type (WT) mice but not in GPRC6A knockout mice [20, 21]. There is no direct evidence proving that uncarboxylated osteocalcin alleviates NAFLD in mice through the GPRC6A signaling pathway. The phenotype of GPRC6A knockout mice demonstrates that this G protein-coupled receptor is involved in the regulation of metabolism, inflammation, and endocrine function. Therefore, we used liver-specific GPRC6A knockout mice to detect whether GPRC6A plays a key role in NAFLD.

## 2. Materials and Methods

### 2.1. Animals and Treatments

GPRC6A flox/flox mice were constructed via Cre-loxP–mediated recombination in mice and purchased from Nanjing Biomedical Research Institute of Nanjing University. To generate liver-specific GPRC6A knockout, GPRC6A flox/flox mice (in which exon 2 of the GPRC6A allele was flanked by loxP sites) were created and backcrossed into the C57BL/6J genetic background. GPRC6A flox/flox and albumin-Cre mice were subsequently produced via intercrossing GPRC6A flox/flox mice with albumin-Cre mice, which express the Cre recombinase transgene under the control of the albumin gene promoter/enhancer elements. Male GPRC6ALKO mice and WT littermates aged 6 to 8 weeks were housed in a specific pathogen-free experimental animal room with a 12-hour light-dark cycle (dark from 7 : 00 p.m. to 7 : 00 a.m.). All experiments conformed to the regulations drafted by the Association for the Assessment and Accreditation of Laboratory Animal Care in Shanghai and were approved by the ethics committee of Shanghai Jiao Tong University Affiliated Sixth People's Hospital (ratification no. 2015-KY-001(T)-(1)).

All mice were given ad libitum access to food and water and were fed with HFD (60% of energy from fat, Research Diets, USA). The mice were injected intraperitoneally with a dose of 30 ng/g recombinant uncarboxylated osteocalcin (Anaspec, USA, catalog number: AS-65307) or with saline solution (vehicle) each day. The stock solution of the recombinant uncarboxylated osteocalcin was prepared by dissolving uncarboxylated osteocalcin powder in DMSO, which concentration was 1 g/mL, then freshly diluted in saline solution at a concentration of 3 ng/*μ*L for intraperitoneal injection [12]. Male GPRC6A^LKO^ mice were randomly divided into the uncarboxylated osteocalcin treatment group (GPRC6A^LKO^ : HFD + OCN, *n* = 8) and the vehicle group (GPRC6A^LKO^ : HFD + NS, *n* = 6). WT littermates were also randomly divided into the uncarboxylated osteocalcin treatment group (WT : HFD + OCN, *n* = 7) and the vehicle group (WT : HFD + NS, *n* = 6).

During the experiment, body weight was monitored weekly with an electronic balance. After 14 hours of overnight fasting, the fasting blood glucose (FBG) levels in the tail vein blood were measured biweekly using an ACCU-CHECK glucometer (Roche, Germany). After 16 weeks of HFD feeding, the mice were killed by euthanasia (1% pentobarbital sodium, 10 *μ*l/g body weight). A portion of the liver was fixed in 4% neutral paraformaldehyde for histological examination, and the other liver tissues were immediately frozen in liquid nitrogen. The tissues were transferred to a −80°C freezer for further analysis.

### 2.2. Intraperitoneal Glucose and Insulin Tolerance Tests

Intraperitoneal glucose tolerance test (IPGTT) and intraperitoneal insulin tolerance test (IPITT) were performed after 16 weeks of HFD feeding. For the IPGTT, after an overnight fast of 14 hours, the mice were injected intraperitoneally with 1 g/kg glucose, and then tail vein blood glucose levels were measured at the indicated time points. For the IPITT, after a fast of 5 hours in the morning, a 1 U/kg dose of insulin (Eli Lilly Inc., USA) was injected intraperitoneally, and the tail vein blood was collected to measure the glucose levels at the indicated time points.

### 2.3. Liver Histological Examination and Liver Triglyceride Measurement

Hematoxylin-eosin (H&E) staining and Oil Red O staining were performed according to the method described previously [22]. Briefly, a piece of the liver was fixed in 4% neutral paraformaldehyde for 24 hours, embedded in paraffin, and subsequently cut into 5 *μ*m-thick sections. Finally, the sections were stained with H&E (Baso, China) and then observed under a microscope (Zeiss, Germany). For Oil Red O staining, the liver tissues were removed from the 4% neutral paraformaldehyde, immersed in sucrose solution for dehydration, and embedded in Tissue-Tek OCT Compound (Sakura Finetek, USA). Subsequently, they were quick-frozen on the freezing platform and cut into 10 *μ*m-thick slices. The slices were stained with Oil Red O (Sigma, USA) for 15 min and then observed under a white light microscope. For measurement of hepatic triglycerides (TGs), 40–50 mg of liver tissue was homogenized in 0.5 ml PBS. After sufficient mixing of 0.4 ml homogenates with 1.6 ml of CHCl3-CH3OH (2 : 1, v/v), the suspension was centrifuged at 3,000 r.p.m. for 10 min at room temperature. The lower organic phase was transferred and air-dried overnight in a chemical hood. The residual liquid was resuspended in 800 *μ*l of 1% Triton X-100 in absolute ethanol, and the concentration of TGs was determined using the serum triglyceride determination kit (Sigma, triglyceride reagent T2449 and free glycerol reagent F6428).

### 2.4. Quantitative Real-Time Polymerase Chain Reaction

Total hepatic RNA was extracted from frozen tissues by using TRIZOL reagent (Life Technologies, USA). cDNA was synthesized from the total RNA by using the PrimeScript^TM^ RT Master Mix kit (Takara, Japan), and real-time PCR was performed with the SYBR^®^ Premix Ex Taq^TM^ II kit. The relative expression of the target genes was normalized to the expression of actin beta (ACTB). The mRNA expression of ACTB, stearoyl-coenzyme A desaturase 1 (SCD1), sterol regulatory element-binding transcription factor 1 (SREBP1), diacylglycerol O-acyltransferase 1 (DGAT1), cell death-inducing DNA fragmentation factor alpha subunit-like effector A (CIDEA), acyl-coenzyme A oxidase 1 (ACOX1), peroxisome proliferator-activated receptor alpha (PPARA), acyl-coenzyme A dehydrogenase medium chain (ACADM), peroxisome proliferative-activated receptor gamma coactivator 1 alpha (PPARGC1A), chemokine (C-C motif) ligand 2 (CCL2), tumor necrosis factor (TNF), activating transcription factor 6 (ATF6), X-box binding protein 1 (XBP1), heat shock protein 5 (HSPA5), and glucose-6-phosphatase catalytic subunit (G6PC) was analyzed (Table 1).

ACADM, acyl-coenzyme A dehydrogenase medium chain; ACOX1, acyl-coenzyme A oxidase 1; ATF6, activating transcription factor 6; CIDEA, cell death-inducing DNA fragmentation factor alpha subunit-like effector A; CCL2, chemokine (C-C motif) ligand 2; DGAT1, diacylglycerol O-acyltransferase 1; G6PC, glucose-6-phosphatase catalytic subunit; HSPA5, heat shock protein 5; PPARGC1A, peroxisome proliferator-activated receptor gamma coactivator 1 alpha; PPARA, peroxisome proliferator-activated receptor alpha; SCD1, stearoyl-coenzyme A desaturase 1; SREBP1, sterol regulatory element-binding transcription factor 1; TNF, tumor necrosis factor; XBP1, X-box binding protein 1.

### 2.5. Western Blot Analysis

Total hepatic protein was isolated from frozen tissues by RIPA lysis buffer which was mixed with protease inhibitors. The mixture was centrifuged at 4°C for 10 min, and the supernatant solution was collected. After the proteins were quantified, equal amounts of protein were used for SDS-PAGE electrophoresis and then transferred to PVDF membranes (Millipore, USA). The membranes were incubated with a primary antibody against GPRC6A (Santa Cruz Biotechnology, USA) at 4°C overnight following blocking with 5% milk and then incubated with corresponding horseradish peroxidase-conjugated secondary antibodies (Cell Signaling Technology, USA) at room temperature for 1 hour. The protein strips were visualized by using an enhanced chemiluminescent kit (GE Healthcare, USA) and an Image Quant LAS 4000 mini Gel Scanner (GE Healthcare, USA). The protein bands were quantified using ImageJ software (National Institutes of Health, USA) and normalized to *ß*-actin (Abcam, UK) for analysis.

### 2.6. Statistical Analysis

All data are expressed as the mean ± standard error of the mean (SEM), and analysis were performed SPSS version 22.0 software (SPSS Inc., USA). *T*-tests were performed between two groups, and *P* values were two-tailed with the statistical significance set at 0.05.

## 3. Results

### 3.1. The Effects of Osteocalcin and GPRC6A^LKO^ on Body Weight and Fasting Blood Glucose

**Figure 1:**
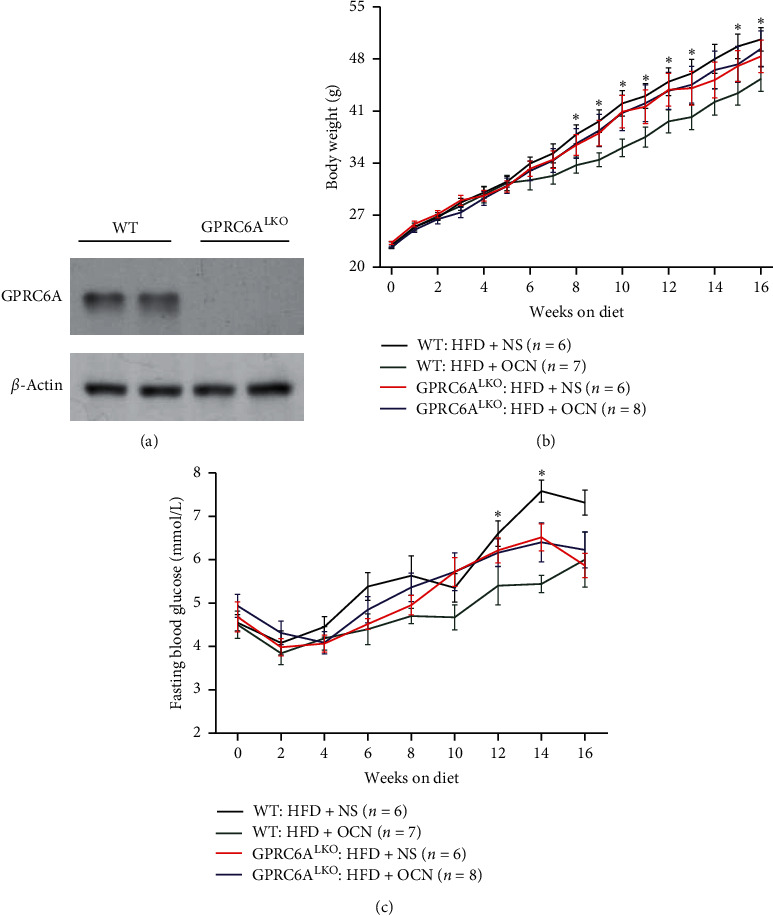
The effects of osteocalcin and GPRC6ALKO on body weight and fasting blood glucose. (a) Verification of GPRC6ALKO through western blotting; (b) body weight (g); (c) fasting blood glucose levels (mmol/L). GPRC6ALKO mice or WT mice were fed with HFD diet and treated daily with 30 ng/g/day uncarboxylated osteocalcin or saline solution. Data were expressed as the mean ± SEM and were analyzed by independent-samples *T*-test. *∗P* < 0.05, the WT : HFD + OCN group compared with the WT : HFD + NS group. GPRC6ALKO, liver-specific conditional GPRC6A knockout; WT, wild-type; NS, saline solution; HFD, high-fat diet; OCN, osteocalcin. The effects of osteocalcin and GPRC6ALKO on glucose tolerance and insulin sensitivity.

At the beginning of the experiments, we verified that GPRC6A was conditionally knocked out in the liver through western blotting (Figure 1(a)). During the experiments, body weight was monitored weekly and FBG was detected biweekly. From the 8^th^ to the 13^th^ weeks and the 15^th^ to the 16^th^ weeks, the WT : HFD + OCN group had significantly lower body weight than the WT : HFD + NS group (all *P* < 0.05, Figure 1(b)). There were no significant differences in body weight between the WT : HFD + NS group and the GPRC6A^LKO^ : HFD + NS group (all *P* > 0.05), which suggested that body weight was not significantly influenced by GPRC6A^LKO^. There were also no significant differences in body weight between the GPRC6A^LKO^ : HFD + OCN group and the WT : HFD + OCN group (all *P* > 0.05). During the 12^th^ and the 14^th^ weeks, the WT : HFD + OCN group had significantly lower FBG levels than the WT : HFD + NS group (all *P* < 0.05) (Figure 1(c)), while without treatment of uncarboxylated osteocalcin, there were no significant differences between the GPRC6A^LKO^ : HFD + NS group and the WT : HFD + NS group.

**Figure 2:**
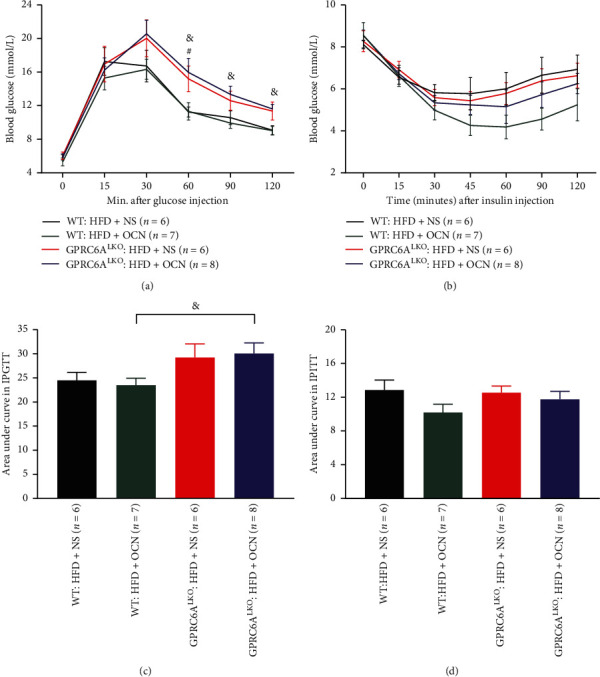
The effects of osteocalcin and GPRC6ALKO on glucose tolerance and insulin sensitivity. (a), (c) Blood glucose levels in each time point and the area under curves in the intraperitoneal glucose tolerance test. (b), (d) Blood glucose levels in each time point and the area under curves in the intraperitoneal insulin tolerance test. Data were expressed as the mean ± SEM and were analyzed using independent-samples *T*-test. ^#^*P* < 0.05, the GPRC6ALKO : HFD + NS group compared with the WT : HFD + NS group; ^&^*P* < 0.05, the GPRC6ALKO : HFD + OCN group compared with the WT : HFD + OCN group. GPRC6ALKO, liver-specific conditional GPRC6A knockout; WT, wild-type; NS, saline solution; HFD, high-fat diet; OCN, osteocalcin. The effects of osteocalcin and GPRC6ALKO on HFD-induced hepatic steatosis.

Sixteen weeks after daily treatment with uncarboxylated osteocalcin or vehicle, the IPITT and IPGTT were conducted. As expected, the GPRC6ALKO : HFD + OCN group had significantly worse glucose tolerance than the WT : HFD + OCN group (Figures 2(a) and 2(c)), although there was no significant difference in insulin sensitivity (Figures 2(b) and 2(d)). It suggested that GPRC6A played a key role in regulating glucose homeostasis.

**Figure 3:**
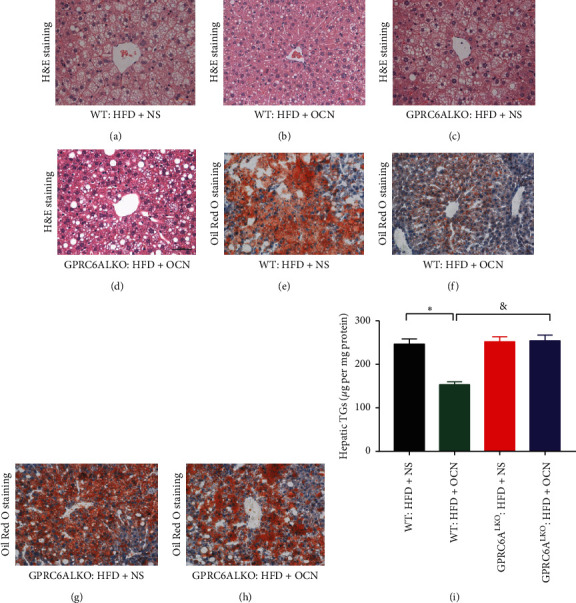
The effects of osteocalcin and GPRC6ALKO on HFD-induced hepatic steatosis. H&E staining (×400) and Oil Red O staining (×200) were performed in the liver tissues: (a), (e) WT : HFD + NS; (b), (f) WT : HFD + OCN; (c), (g) GPRC6ALKO : HFD + NS; (d), (h) GPRC6ALKO : HFD + OCN; (i) liver content of TGs. In Figure 3(i), data were expressed as the mean ± SEM and were analyzed by independent-samples *T*-test. ^*∗*^*P* < 0.05, the WT : HFD + OCN group compared with the WT : HFD + NS group. ^&^*P* < 0.05, the GPRC6ALKO : HFD + OCN group compared with the WT : HFD + OCN group. GPRC6ALKO, liver-specific conditional GPRC6A knockout; WT, wild-type; NS, saline solution; HFD, high-fat diet; OCN, osteocalcin.

HFD-induced hepatic steatosis was less serious in the WT : HFD + OCN group than that in the WT : HFD + NS group, which suggested that uncarboxylated osteocalcin treatment remarkably attenuated HFD-induced hepatic steatosis (Figures 3(a), 3(b), 3(e), 3(f), and 3(i)). The pathological changes of HFD-induced NAFLD were also more serious in the GPRC6ALKO : HFD + OCN group than that of the WT : HFD + OCN group (Figure 3(b), 3(d), 3(f), 3(h), and 3(i)), which suggested that uncarboxylated osteocalcin did not protect GPRC6ALKO mice from HFD-induced hepatic steatosis.

### 3.2. Exogenous Osteocalcin Treatment Alters Gene Expression in HFD-Induced Hepatic Steatosis

**Figure 4:**
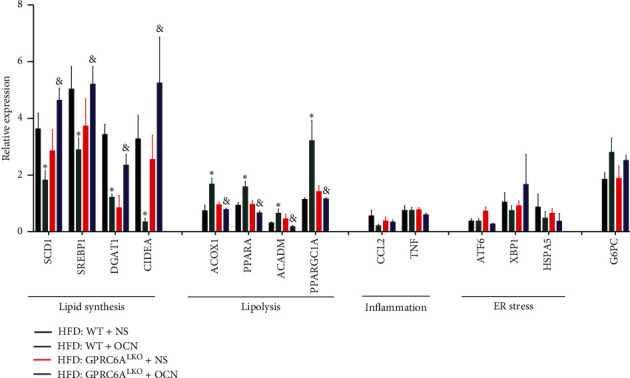
Relative expression of genes involved in lipid synthesis, lipolysis, inflammation, ER stress, and gluconeogenesis. Data were expressed as the mean ± SEM and were analyzed by independent-samples *T*-test. *∗P* < 0.05, HFD : WT + OCN compared with HFD : WT + NS; ^&^*P* < 0.05, the GPRC6ALKO : HFD + OCN group compared with the WT : HFD + OCN group. ACADM, acyl-coenzyme A dehydrogenase medium chain; ACOX1, acyl-coenzyme A oxidase 1; ATF6, activating transcription factor 6; CIDEA, cell death-inducing DNA fragmentation factor alpha subunit-like effector A; CCL2, chemokine (C-C motif) ligand 2; DGAT1, diacylglycerol O-acyltransferase 1; G6PC, glucose-6-phosphatase catalytic subunit; HSPA5, heat shock protein 5; PPARGC1A, peroxisome proliferator-activated receptor gamma coactivator 1 alpha; PPARA, peroxisome proliferator-activated receptor alpha; SCD1, stearoyl-coenzyme A desaturase 1; SREBP1, sterol regulatory element binding transcription factor 1; TNF, tumor necrosis factor; XBP1, X-box binding protein 1.

We next examined how hepatocyte GPRC6A inactivation modulates the progression of HFD-induced hepatic steatosis. The analysis of hepatic gene expression indicated that the mRNA levels of key genes involved in lipogenesis (SCD1, SREBP1, DGAT1, and CIDEA) were signiﬁcantly decreased and that the mRNA levels of genes involved in lipolysis (ACOX1, PPARA, ACADM, and PPARGC1A) were signiﬁcantly increased in the WT : HFD + OCN group compared with the WT : HFD + NS group (all *P* < 0.05). However, SCD1, SREBP1, DGAT1, and CIDEA were signiﬁcantly higher and PPARA, ACOX1, ACADM, and PPARGC1A were signiﬁcantly lower in the GPRC6ALKO : HFD + OCN group compared with the WT : HFD + OCN group (all *P* < 0.05). Genes involved in inflammation (CCL12 and TNF), ER stress (ATF6, XBP1, and HSPA5), and gluconeogenesis (G6PC) showed no significant differences between these three groups (all *P* > 0.05) (Figure 4). Together, these results demonstrate that GPRC6A is required for alleviating lipogenic activation and HFD-induced hepatic steatosis upon uncarboxylated osteocalcin treatment.

## 4. Discussion

In the present study, a liver-specific conditional GPRC6A knockout mouse model was used to explore the potential mechanisms underlying the ability of uncarboxylated osteocalcin to alleviate NAFLD. For the first time, our results found that uncarboxylated osteocalcin alleviates NAFLD in mice through the GPRC6A signaling pathway. Our study expands the spectrum of functions of GPRC6A, adds further evidence to the concept that the bone and liver regulate each other, and suggests that the pathogenesis of some degenerative diseases of energy metabolism may be more complex than anticipated.

The skeleton is constantly destroying and regenerating itself through bone remodeling. Constant bone remodeling makes skeleton an energy-expensive organ [23]. One might question whether this energy-expensive process is worth it. In recent decades, crosstalk between bone remodeling and energy metabolism has been recognized, providing rationality for the high energy consumption of this process. Osteocalcin is one of the most important hormones involved in the crosstalk between bone remodeling and energy metabolism. Osteocalcin is a bone matrix protein. The high-profile associations between osteocalcin and energy metabolism are mainly based on the uncarboxylated form of osteocalcin. Osteocalcin-deficient mice are hyperglycemic and have lower *ß*-cell mass and decreased energy expenditure [11]. In contrast, treatment with uncarboxylated osteocalcin increases serum insulin levels and improves glucose tolerance and insulin sensitivity [24]. ESP is a gene that is expressed in osteoblasts and negatively regulates the uncarboxylation of osteocalcin. ESP-deficient mice exhibit decreased fat content in the liver [11]. Gupte et al. found that uncarboxylated osteocalcin inhibits nonalcoholic steatohepatitis development [13]. Our previous study also found that osteocalcin improves NAFLD by activating the Nrf2 pathway to alleviate oxidative stress and inhibit the JNK pathway [12].

GPRC6A, as a receptor of multiple ligands, serves as a point of integration of diverse extracellular signals and cellular responses in multiple tissues. GPRC6A is widely expressed in the brain, kidney, testis, pancreas, liver, adipose tissue, etc. [25]. GPRC6A is the putative receptor of osteocalcin. Our current understanding of its tissue-speciﬁc functions largely comes from an analysis of the effects of osteocalcin treatment and mouse genetic studies [14]. Uncarboxylated osteocalcin acts through GPRC6A to increase pancreatic *ß*-cell number, insulin release, and insulin sensitivity [19]. Uncarboxylated osteocalcin induces testosterone production by the testes via GPRC6A [26] and increases the expression of adiponectin in adipocytes via GPRC6A [17]. However, whether GPRC6A in the liver is involved in the potential mechanisms by which osteocalcin alleviates NAFLD remains unclear. In the present study, we constructed a liver-specific GPRC6A knockout mouse model. The HFD-induced hepatic steatosis in GPRC6A^LKO^ mice was more serious than that in WT mice. Upon treatment with uncarboxylated osteocalcin, WT mice were protected from HFD-induced NAFLD while GPRC6A^LKO^ mice still showed obvious hepatic steatosis. The results suggest that GPRC6A plays a key role in NAFLD. Our data show that GPRC6A mediates responses to uncarboxylated osteocalcin treatment in the liver in vivo, mainly through enhanced lipolysis and decreased lipogenesis.

NAFLD is characterized by excess ectopic fat accumulation in the liver. The biological activity of lipid synthesis and lipolysis reveals pathogenic changes in NAFLD. SCD1 is the rate-limiting enzyme in the synthesis of monounsaturated fats. SREBP1 is the key transcription regulation factor for fat synthesis [27]. DGAT1 catalyzes the final step of triglyceride synthesis [28]. CIDEA plays an essential role in generating lipid droplets; thus, CIDEA can be used as a sensitive marker for lipid accumulation [29]. All these genes involved in lipid synthesis were downregulated in uncarboxylated osteocalcin-treated WT mice and were highly expressed in uncarboxylated osteocalcin-treated GPRC6A^LKO^ mice. The opposite was found for lipolysis genes such as ACOX1 (which catalyzes the oxidation of long branched fatty acids) [30], PPARA/PPARGC1A (which promote fatty acid oxidation) [31], and ACADM (which catalyzes the oxidation of medium chain fatty acids) [32]. The above results reveal that GPRC6A mediates the effects of osteocalcin in NAFLD by inhibiting lipid synthesis and promoting lipolysis. Previous studies have found that GPRC6A directly acts on many organs and activates the same signaling pathways, including the ERK, cAMP, PI3K/Akt/mTOR, and AMPK pathways [20, 21, 33, 34]. In the liver, these pathways may also mediate the effects of GPRC6A on inhibiting lipid synthesis and promoting lipolysis. Besides, recently one study showed that osteocalcin can also control fat absorption in intestine [35]. Further studies are needed to clarify the potential mechanisms by which GPRC6A activation alleviates NAFLD in the liver.

## 5. Conclusions

The present study provides evidence that GPRC6A directly mediates the effects of uncarboxylated osteocalcin in alleviating HFD-induced NAFLD in mice. The novel finding of this study expands our knowledge on the role of GPRC6A in energy metabolism and reveals that liver GPRC6A may be a potential target for treating NAFLD.

## Figures and Tables

**Table 1:** Primer list of real-time PCR.

Gene name	Forward (5′-3′)	Reverse (5′-3′)
ACTB	CCACAGCTGAGAGGGAAATC	AAGGAAGGCTGGAAAAGAGC
SCD1	TTCTTCTCTCACGTGGGTTG	CGGGCTTGTAGTACCTCCTC
SREBP1	GGAGCCATGGATTGCACATT	GGCCCGGGAAGTCACTGT
DGAT1	GATTGTGGGCCGATTCTTCC	CATACATGAGCACAGCCACC
CIDEA	ATCACAACTGGCCTGGTTACG	TACTACCCGGTGTCCATTTCT
ACOX1	GCCTGAGCTTCATGCCCTCA	ACCAGAGTTGGCCAGACTGC
PPARA	GGGCAGAGCAAGTCATCTTC	CCTCTGGAAGCACTGAGGAC
ACADM	GATCGCAATGGGTGCTTTTGATAGAA	AGCTGATTGGCAATGTCTCCAGCAAA
PPARGC1A	AAGAGCGCCGTGTGATTTAC	ACGGTGCATTCCTCAATTTC
CCL2	CAGCCAGATGCAGTTAACGC	GCCTACTCATTGGGATCATCTTG
TNF	CGTCAGCCGATTTGCTATCT	CGGACTCCGCAAAGTCTAAG
ATF6	AACTGTTCCCTGGACAGCAC	TTCTCCTTGGCACCCATTAG
XBP1	GGCATCTCAAACCTGCTTTC	TCCCAGGAGTGGTCTGTACC
HSPA5	CAGATCTTCTCCACGGCTTC	GCAGGAGGAATTCCAGTCAG
G6PC	CCTCCTCAGCCTATGTCTGC	AACATCGGAGTGACCTTTGG

## Data Availability

The data used to support the findings of this study are available from the corresponding authors upon request.
